# Multifactorial Modeling of Impairment of Evoked Gamma Range Oscillations in Schizophrenia

**DOI:** 10.3389/fncom.2016.00089

**Published:** 2016-08-26

**Authors:** Christoph Metzner, Achim Schweikard, Bartosz Zurowski

**Affiliations:** ^1^Biocomputation Research Group, University of HertfordshireHatfield, UK; ^2^Institute for Robotics and Cognitive Systems, University of LuebeckLuebeck, Germany; ^3^Centre for Integrative Psychiatry, University of LuebeckLuebeck, Germany

**Keywords:** auditory entrainment, schizophrenia, computational model, oscillations, circuit abnormalities, parameter search, multifactoriality

## Abstract

Despite a significant increase in efforts to identify biomarkers and endophenotypic measures of psychiatric illnesses, only a very limited amount of computational models of these markers and measures has been implemented so far. Moreover, existing computational models dealing with biomarkers typically only examine one possible mechanism in isolation, disregarding the possibility that other combinations of model parameters might produce the same network behavior (what has been termed “multifactoriality”). In this study we describe a step toward a computational instantiation of an endophenotypic finding for schizophrenia, namely the impairment of evoked auditory gamma and beta oscillations in schizophrenia. We explore the multifactorial nature of this impairment using an established model of primary auditory cortex, by performing an extensive search of the parameter space. We find that single network parameters contain only little information about whether the network will show impaired gamma entrainment and that different regions in the parameter space yield similar network level oscillation abnormalities. These regions in the parameter space, however, show strong differences in the underlying network dynamics. To sum up, we present a first step toward an *in silico* instantiation of an important biomarker of schizophrenia, which has great potential for the identification and study of disease mechanisms and for understanding of existing treatments and development of novel ones.

## 1. Introduction

Over the last years, the traditional diagnostic classifications used in psychiatry have been questioned and a breakdown into simpler categories like endophenotypes or cognitive domains has been proposed (Leboyer et al., 1998; Braff et al., 2007; Nuechterlein et al., 2008; Insel et al., 2010; Cuthbert and Insel, 2013). This is mainly due to the fact that the gap between symptom-based classifications on the one hand and genes and molecules on the other hand is huge and a clear mapping in between not in sight. For example, single susceptibility genes for most disorders have only small predictive value regarding phenotype (Flint and Munafò, 2007; Need et al., 2009; Cirulli et al., 2010). Underlying these new proposals is the hope that the simpler categories will map nicely to alterations at the genetic/molecular level (e.g., Meyer-Lindenberg and Weinberger, 2006). This effort has mainly focused on the identification and validation of biomarkers for psychiatric disorders using *in vivo* and *in vitro* studies. As Siekmeier (2015) argues, computational modeling approaches are ideally suited to complement these efforts in order to construct biomarker based models of psychiatric disorders for two reasons: (1) Models can allow for an identification and a mechanistic understanding of illness mechanisms. Not only is “*in silico*” testing of such models easier and cheaper than human or animal studies, they also offer the great advantage of making all available variables and assumptions explicit and accessible. (2) Computational models can be used very effectively for the development of new neuropsychiatric drugs (e.g., Siekmeier and vanMaanen, 2013). However, most modeling efforts in computational psychiatry focus on the study of a single potential mechanism in isolation, as recently pointed by Pavão et al. (2015). They demonstrate nicely that the same network behavior can be produced by a huge number of different instances of a neural network, i.e., that network dynamics is multifactorial by nature, using two established models of schizophrenia. This suggests that, in order to understand the mechanisms underlying psychiatric disorders like schizophrenia, it is not enough to discover the genetic alterations but that it is also essential to understand how these alterations interact in order to give rise to endophenotypical and behavioral changes. Computational models offer a unique possibility to address this need.

Here, we focus on modeling deficits in schizophrenic patients. Over the last more than 50 years it has become clear that many different neurotransmitter systems are implicated in the neuropathology of schizophrenia. This started with the dopamine hypothesis (e.g., Carlsson and Lindqvist, 1963) and the implication of serotonin (e.g. Woolley and Shaw, 1954) in the early 50 and 60 s, moving on to the glutamate hypothesis (e.g., Javitt et al., 1994; Javitt, 2004) followed by an implication of GABAergic circuits (e.g., Perry et al., 1979; Rosso et al., 2006). This implication of several important neurotransmitter systems makes it clear that the interactions of these systems are of paramount importance for an understanding of the heterogeneity and complexity of schizophrenia, for which the integrative computational framework outlined above can be an ideal tool.

In this study we present a step toward an *in silico* model of an endophenotypic biomarker of schizophrenia that explores the multifactorial nature of the underlying network. We focus on abnormal gamma rhythms in the auditory system, since very convincing evidence for abnormalities in this frequency band has accumulated over the last decades (see Siekmeier, 2015), It has been proposed that oscillations in and between circuits underlie efficient communication of ensembles and routing of information in the brain (“Communication through coherence,” Fries, 2005), with gamma frequency oscillations playing an important role (Bastos et al., 2015). Although this concept has been critiqued by some authors (e.g., Ray and Maunsell, 2010; Ray et al., 2013; Buzsáki and Schomburg, 2015), there is consensus that neural oscillations at least constitute a signature of the underlying computations performed in the circuit.

It has been reported that schizophrenic patients show multiple alterations in the gamma rhythm in different experimental paradigms, not only in the auditory system (Spencer et al., 2003; Uhlhaas and Singer, 2010). In the auditory system, however, they are particularly prominent (Light et al., 2006; Spencer et al., 2008) and linked with auditory hallucinations (Spencer et al., 2009). Krishnan et al. (2009) report decreases in EEG power in a steady-state auditory evoked potential (SSAEP) task, using amplitude-modulated tones, specific to the 40–50 Hz range. Kwon et al. (1999) showed reduced EEG power in the gamma frequency range for schizophrenic patients compared with healthy controls in a click entrainment paradigm. This has been replicated using the same paradigm in an MEG study by Vierling-Claassen et al. (2008). While Krishnan et al. (2009) report no significant changes in the beta range for amplitude-modulated tones, both click entrainment studies also show alterations in the low beta range (at around 20 Hz), although less pronounced. On the other hand, multiple circuit abnormalities have been described in schizophrenia: (i) reduced reduced somal size, spine density, and dendritic field size on pyramidal cells (Garey et al., 1998; Glantz and Lewis, 2000; Pierri et al., 2001; Broadbelt et al., 2002; Chana et al., 2003; Black et al., 2004; Sweet et al., 2008), (ii) reduced synaptophysin levels (Perrone-Bizzozero et al., 1996; Glantz and Lewis, 1997), (iii) decreased expression of genes encoding synaptic proteins (Mirnics et al., 2000; Torrey et al., 2005), (iv) decreases in GAD67 expression (an enzyme responsible for GABA synthesis) (Akbarian et al., 1995), and (v) hypoactivation of NMDA receptors at inhibitory interneurons (Kantrowitz and Javitt, 2010).

In summary, converging experimental evidence suggests a deficit in maintaining gamma rhythms in the auditory system of schizophrenic patients. Whereas deficits in the gamma range are most prominent, there is inconsistent evidence of changes in the beta range as well. Several different mechanisms have been shown to able produce selective reductions in gamma entrainment in models of the auditory cortex. However, given the likely multifactorial nature of disorders like schizophrenia, as we argued above, a more profound investigation of the interplay of these identified circuit mechanisms is needed. Furthermore, a more detailed exploration of subtle differences in the beta range might shed further light on the contributions of these mechanisms.

In this study we explored possible mechanisms underlying deficits in gamma range auditory entrainment in schizophrenia using a biophysically detailed neural network model. Notably, our approach differs from the above-mentioned, except for the study by Siekmeier and vanMaanen (2013), in that we do not restrict our analysis to one possible mechanism but rather the multifactorial nature of the relationship between cellular level abnormalities and endophenotypic measures, i.e., we explore the parameter space of possible circuit abnormalities that might give rise to SZ-like oscillatory behavior. In particular, we tested four hypotheses:

- *Hypothesis I*: Given the results of earlier studies (Pavão et al., 2015; Mäki-Marttunen et al., 2016), we hypothesized that SZ-like behavior would be produced by combinations of parameters rather than being primarily caused by a single parameter. Thus, we expected the information content of single parameters regarding the “computational network phenotype” to be low.
- *Hypothesis II*: We hypothesized that the exact definition of SZ-like (i.e., the number of included features, such as power decrease at 40 Hz, power increase at 20 Hz) would have a strong influence on the location in parameter space of regions producing SZ-like network behavior (hereafter, “SZ-regions”).
- *Hypothesis III*: We expected to find differences regarding the underlying network dynamics that produce SZ-like behavior between SZ-regions.

## 2. Materials and methods

### 2.1. Computational model

The auditory cortex model is a slightly downscaled version of the model described by Beeman (2013) (for a summary of the most important network parameters see Table 1). Instead of the 48 × 48 excitatory and 24 × 24 inhibitory neurons of the original model, we used a smaller version having 24 × 24 excitatory and 12 × 12 inhibitory neurons. The reduction in size yielded a significant speedup of simulations and no qualitative effect on the simulated EEG power spectra. The excitatory cells are based on the pyramidal cell model of Bush and Sejnowski (1993), containing voltage and calcium activated channels, and there are dual-exponential synaptically activated channels at appropriate locations on the dendrites (see Beeman, 2013). The basket cell is modeled simply with a soma and a single cylindrical dendrite. The active channels used in the soma are a small set of modified hippocampal CA3 region channels (Traub et al., 1994) with parameters adapted to yield behavior typical of neocortical cells.

**Table 1:** **Connection probabilities ***p***, conductance weights ***g***, and synaptic time constants (τ_**1**_ rise time, τ_**2**_ decay time)**.

	**Receiving cell**	
	**Excitatory cell**	**Inhibitory cell**
**Sending cell**		
Excitatory cell	*p_ee_*/*g_ee_*/τ_1_/τ_2_ = 0.15/30*nS*/1 *ms*/3 *ms*	*p_ei_*/*g_ei_*/τ_1_/τ_2_ = 0.45/0.1*nS*/3 *ms*/3 *ms*
Inhibitory cell	*p_ie_*/*g_ie_*/τ_1_/τ_2_ = 0.6/0.6*nS*/1 *ms*/6 *ms*	*p_ii_*/*g_ii_*/τ_1_/τ_2_ = 0.6/0.15*nS*/1 *ms*/6 *ms*
Thalamic input	*p_ie_*/*g_ie_*/τ_1_/τ_2_ = 1.0/50*nS*/1 *ms*/3 *ms*	*p_ii_*/*g_ii_*/τ_1_/τ_2_ = 0.65/1.5*nS*/3 *ms*/3 *ms*
Background noise	*p_ie_*/*g_ie_*/τ_1_/τ_2_ = 1.0/90*nS*/1 *ms*/3 *ms*	*p_ii_*/*g_ii_*/τ_1_/τ_2_ = −/−/−/−

Connectivity in the network was random, with the probability for connections decreasing exponentially with radial distance. Synaptic weights, however, were fixed independent of distance (see recent experiments Yuan et al., 2011; Levy and Reyes, 2012).

In addition to the interconnections between the cells, the excitatory cells receive a Poisson-distributed random activation at their basal dendrites in order to represent excitatory inputs from other layers. The default parameters and average frequency were chosen in order to give background levels of firing for the two populations in agreement with those measured by Steriade et al. (2001).

The model was implemented and run in Genesis (Bower and Beeman, 1998; release 2.3). Integration was performed using the Crank-Nicholson method with a time step of 0.00002 ms. The network was simulated for 10 s in each simulation run.

### 2.2. Implementation of click entrainment

For the auditory click entrainment at a certain frequency ω, we calculated spike trains using a Poisson process with a rate ω. Each cell (excitatory and inhibitory) received such a spike train at the excitatory synapse where the afferent thalamic input arrives. For the excitatory pyramidal cell this synapse is located at its proximal apical dendrite and for the basket cell at the only dendritic segment. EEG and MEG studies have revealed a click entrainment deficit in schizophrenic patients at 40 Hz but intact entrainment at 30 Hz (Kwon et al., 1999; Vierling-Claassen et al., 2008). We therefore stimulated at 30 and 40 Hz.

### 2.3. Implementation of cellular/network level abnormalities

We implemented three different types of impairments on the cellular and network levels: (1) reduced inhibitory connectivity, (2) changed inhibitory output and, (3) prolonged GABAergic decay times. Table 2 summarizes the changes made and gives the total number of simulations conducted.

**Table 2:** **Implementation of cellular/network level abnormalities in the parameter space search**.

	**Inhibitory connectivity**		**Inhibitory output**		**GABAergic decay times**	
	***n_ie_***	***n_ii_***	***w_ie_***	***w_ii_***	***τ_ie_***	***τ_ii_***
Change	100%, 75%,	100%, 75%,	150%, 125%, 100%,	150%, 125%, 100%,	6 ms, 15 ms,	6 ms, 15 ms,
	50%	50%	75%, 50%	75%, 50%	25 ms	25 ms
Total	3·3·5·5·3·3 = 2025 Simulations					

#### 2.3.1. Reduced inhibitory connectivity

We reduced the number *n*_*ie*_ of inhibitory connections to excitatory cells and the number *n*_*ii*_ of inhibitory connections to inhibitory cells. We reduced the number of connections in two steps (to 75 and 50% of its original value) for each connection type independently.

#### 2.3.2. Changed inhibitory output

We reduced the output of the inhibitory neurons by changing the weights *w*_*ie*_ and *w*_*ii*_ of the inhibitory connections to excitatory cells and of the inhibitory connections to inhibitory cells, respectively. Here we not only reduced the weights to 75 and 50% but also increased them to 125 and 150% (see also the computational study of Siekmeier and vanMaanen, 2013), because there is evidence of post-synaptic upregulation as a compensatory means for a reduced GABAergic tone due to reduced connectivity (Lisman et al., 2008).

#### 2.3.3. Prolonged GABAergic decay times

Last, we also modified the decay time constants at GABAergic synapses, since it has been shown that they have a significant effect on auditory click entrainment (Vierling-Claassen et al., 2008; Vierling-Claassen and Kopell, 2009). We chose to increase the decay time constants τ_*ie*_ and τ_*ii*_ at inhibitory synapses (on excitatory and inhibitory cells, respectively) independently of each other to 15 and 25 ms.

### 2.4. Analysis methods

#### 2.4.1. Power spectra

We calculated a simulated EEG signal by summing all excitatory postsynaptic currents (EPSCs) occurring at excitatory cells. Power spectra were then computed from this signal using the Fast Fourier Transform (implemented in Python using Scipy's signal processing module).

#### 2.4.2. Identification of “schizophrenic” parameter combinations - “illness metrics”

In order to assess how SZ-like the resulting network behavior of a given parameter combination was, we ran simulations at both driving frequencies (30 and 40 Hz) and calculated the power spectra.

We then identified different parameter vectors displaying a strong SZ-like behavior (details are described below) and compared them against the normal condition, i.e., the network model as described above without any parameter changes. To ensure that we had captured a robust control condition, we simulated 20 different control subjects by changing the seed for the random generator. Changing the random seed yields a network with the same overall connectivity statistics, but with a different actual connectivity (see also Siekmeier and vanMaanen, 2013).

#### 2.4.3. 40 Hz reduction - illness metric I

We identified those parameter vectors that displayed a strong reduction of the 40 Hz component in the 40 Hz drive condition. This reduction seems to be the most prominent change in beta/gamma frequency entrainment in schizophrenic patients (Kwon et al., 1999; Vierling-Claassen et al., 2008; Siekmeier and vanMaanen, 2013).

In order to quantify the reduction of the 40 Hz component, we used the following metric:

$$\begin{array}{l} M1\left(PV\right)=\left(1-\frac{P40_{PV}}{{\bar{P40}}_{Ctrl}}\right). \end{array}$$

Here, *P*40_*PV*_ denotes the 40 Hz component at 40 Hz drive of the given parameter vector and ${\bar{P40}}_{Ctrl}$ denotes the mean 40 Hz component at 40 Hz drive of the 20 control subjects.

#### 2.4.4. 40 Hz reduction and 30 Hz validity - illness metric II

Next, we additionally used the 30 Hz component in response to 30 Hz drive, since, experimental evidence strongly suggests that this component remains intact in patients with schizophrenia (Kwon et al., 1999; Vierling-Claassen et al., 2008). Therefore, we modified the above metric to:

$$\begin{array}{l} M2(PV)=\{\begin{array}{ll} 0 & \text{if}|P30_{PV}-{\bar{P30}}_{Ctrl}|<3\sigma_{Ctrl_{30}}\\ M2\prime & \text{else} \end{array} \end{array}$$

with

$$\begin{array}{l} M2\prime =\left(1-\frac{P40_{PV}}{{\bar{P40}}_{Ctrl}}\right) \end{array}$$

Here, again *P*40_*PV*_ denotes the 40 Hz component at 40 Hz drive of the given parameter vector and ${\bar{P40}}_{Ctrl}$ denotes the mean 40 Hz component at 40 Hz drive of the 20 control subjects. Furthermore, *P*30_*PV*_ denotes the 30 Hz component at 30 Hz drive of the given parameter vector, ${\bar{P30}}_{Ctrl}$ denotes the mean 30 Hz component at 30 Hz drive of the 20 control subjects and σ_*Ctrl*_30__ denotes the standard deviation of the 30 Hz component at 30 Hz drive of the 20 control subjects.

That means, we simply excluded all PVs that showed a deviation from the standard control network, while all remaining PVs received the same score as with metric M1.

#### 2.4.5. 40 Hz reduction and 30 Hz validity and 20 Hz facilitation - illness metric III

In both EEG and MEG studies, differences not only in the power at 40 Hz but also the power at 20 Hz in the 40 Hz drive condition have been found, where patients show an increase in power (Kwon et al., 1999; Vierling-Claassen et al., 2008).

Therefore, we extended M2 to also incorporate this difference:

$$\begin{array}{l} M3(PV)=\{\begin{array}{ll} 0 & \text{if}|P30_{PV}-{\bar{P30}}_{Ctrl}|>3\sigma_{Ctrl_{30}}\\ M3\prime & \text{else} \end{array} \end{array}$$

with

$$\begin{array}{l} M3\prime =\left(\frac{1}{2}\left(1-\frac{P40_{PV}}{{\bar{P40}}_{Ctrl}}\right)-\frac{1}{2}\left(1-\frac{P20_{PV}}{{\bar{P20}}_{Ctrl}}\right)\right). \end{array}$$

Here, *P*40_*PV*_, ${\bar{P40}}_{Ctrl}$, *P*30_*PV*_, and ${\bar{P30}}_{Ctrl}$ are as above. Furthermore, *P*20_*PV*_ denotes the 20 Hz component at 40 Hz drive for the given PV and ${\bar{P20}}_{Ctrl}$ denotes the mean 20 Hz component at 40 Hz drive for the 20 control subjects.

In order to see whether the cellular level parameters were predictive of a “schizophrenic phenotype,” we took an information theory based approach, as outlined in Pavão et al. (2015). That is, we calculated the normalized mutual information (nMI) between our independent variables (denoted by *X*_*p*_, where *p* is one of the six parameters τ_*IE*_,τ_*II*_,*r*_*IE*_,*r*_*II*_,*w*_*IE*_, and *w*_*II*_) and our dependent variable *Y*_*M*_*i*__ (i.e., the phenotype as indicated by the given metric M_*i*_). We calculated nMI not only for single, isolated parameters but also for all possible combinations of parameters (again see Pavão et al., 2015). Specifically, nMI was calculated by

$$\begin{array}{l} nMI\left(X_{i},\ldots ,X_{j},Y_{M_{i}}\right)=100\cdot \left(MI\left(X_{i},\ldots ,X_{j},Y_{M_{i}}\right)/H\left(Y_{M_{i}}\right)\right), \end{array}$$

where *MI*(*X*_*i*_, …, *X*_*j*_, *Y*_*M*_*i*__) is the mutual information between the parameters *X*_*i*_, …, *X*_*j*_ and the phenotype *Y*_*M*_*i*__ and *H*(*Y*_*M*_*i*__) is the Shannon entropy of *Y*_*M*_*i*__. Mutual information is calculated by

$$\begin{array}{l} MI(X_{i},\ldots ,X_{j},Y_{M_{i}})=H(X_{i},\ldots ,X_{j})\;+\;H(Y_{M_{i}}))\\ \;-\;H(X_{i},\ldots ,X_{j},Y_{M_{i}}), \end{array}$$

where again *H* denotes the Shannon entropy, generally defined as

$$\begin{array}{l} H\left(X,Y\right)=\underset{k}{\sum }\underset{l}{\sum }p\left(x_{k},y_{l}\right)\log_{2}\left(p\left(x_{k},y_{l}\right)\right) \end{array}$$

for two random variables *X* and *Y*.

After investigating the overall parameter space, we went further to explore potential mechanisms underlying gamma entrainment deficits and their dependence on the particular illness metric. Therefore, we used the PVs having the highest M1, M2, and M3 values, respectively, for further analysis (denoted by PV_*M*1_, PV_*M*2_, and PV_*M*3_, respectively). First, we calculated the ratio of “schizophrenia-like” PVs of all PVs showing a high value for M_*i*_(for all three metrics). We calculated for each instance *p*_*i*_ of each parameter *p* (e.g., parameter τ_*ie*_ has three instances *p*_1_ = 6 *ms*, *p*_2_ = 15 *ms*, and *p*_3_ = 25 *ms*) how many percent of the “schizophrenia-like” PVs had this particular parameter instance *p*_*i*_. The higher this percentage, the stronger the influence of this particular parameter instance on the change in oscillatory dynamics. We repeated these calculations three times, where “schizophrenia-like” was defined by metric M1, M2, and M3, respectively.

Next, we wanted to check whether the effect was robust. Therefore, we simulated 20 schizophrenic subjects for each of the three PVs. We then compared the schizophrenic subjects and the control subjects using three mixed model ANOVAs, with GROUP (control, schizophrenic-M_*i*_; with *i* ∈ {1, 2, 3}) as between subjects factor and POWER (40 and 20 Hz power at 40 Hz drive; and 30 Hz power at 30 Hz drive) as a repeated measures factor (similar as in Siekmeier and vanMaanen, 2013).

Finally, we further analyzed the dynamic behavior for the three PVs. To this end, we calculated stimulus-locked EEG signals, where we averaged the EEG signal over two consecutive stimulation cycles, for the 40 Hz drive condition. We also calculated stimulus-locked spike histograms for the pyramidal and the basket cell populations.

## 3. Results

### 3.1. Control network model

The standard network model was able to entrain to both driving frequencies used in our simulations. In order to establish a robust baseline for our further analyses, we simulated 20 different instances of the control network by changing the seed for the random number generator. This yielded different specific cell-to-cell connectivity, however, leaving the overall connection probabilities unchanged.

**Figure 1:**
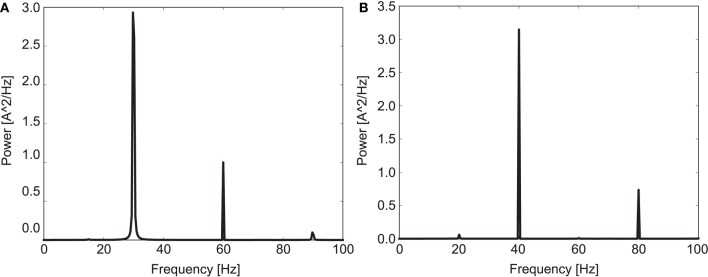
**Mean power spectrum for the 20 control subjects in response to (A) 30 Hz drive and (B) 40 Hz drive**.

Figure 1 shows a power spectrum plot of the simulated EEG signal for both driving frequencies for the 20 control subjects. It is clearly visible that the model shows entrainment to both driving frequencies in agreement with human experimental data (Kwon et al., 1999; Vierling-Claassen et al., 2008). The entrainment presented a robust phenomenon which was hardly influenced by the actual cell-to-cell connectivity for the 30 and 40 Hz drive. Notably, in the control network entrainment is strong at the driving frequency but very weak at other frequencies (especially at 30 Hz drive).

### 3.2. Consequences of circuit abnormalities

#### 3.2.1. Hypothesis I

**Figure 2:**
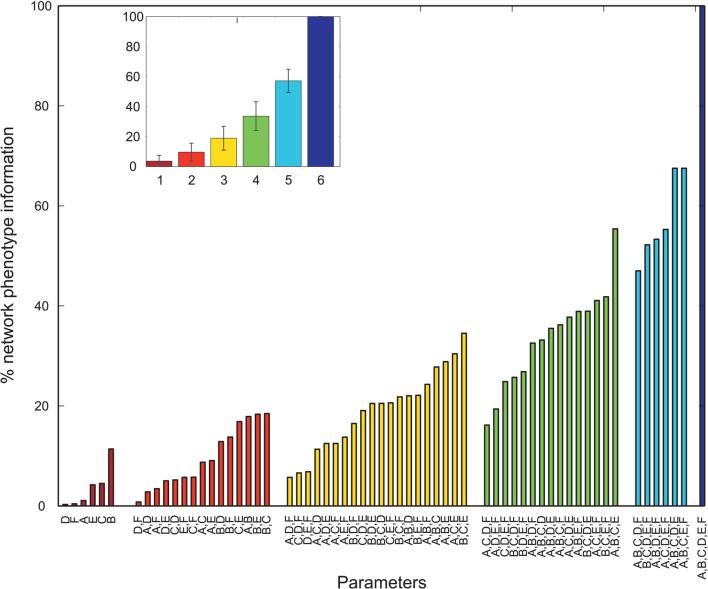
**Mutual information shared by single network parameters or combinations thereof with the phenotype of the network**. Phenotype is determined by metric *M*1. Network Parameters: *A* = Decay time constant at GABAergic I-to-E synapses, *B* = Decay time constant at GABAergic I-to-I synapses, *C* = Reduction of number of GABAergic I-to-E connections, *D* = Reduction of number of GABAergic I-to-I connections, *E* = Change of weight at GABAergic I-to-E connections, *F* = Change of weight at GABAergic I-to-I connections. Inset displays mean information (± standard deviation) for different numbers of parameters.

**Figure 3:**
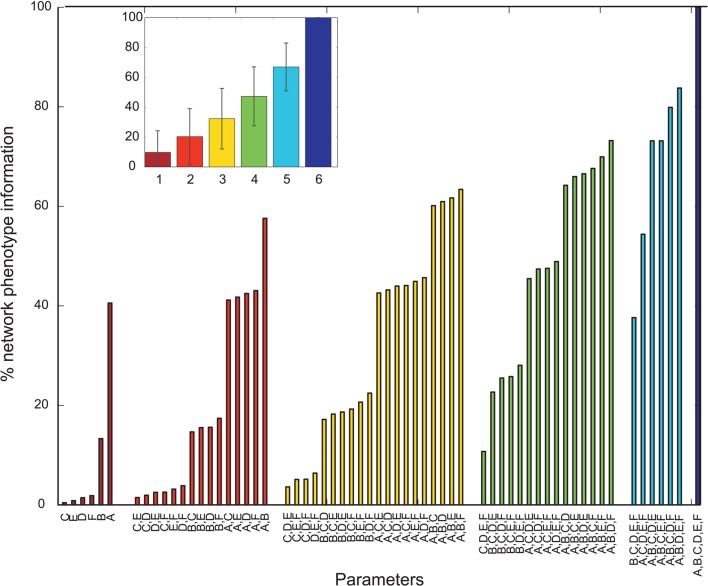
**Mutual information shared by single network parameters or combinations thereof with the phenotype of the network**. Phenotype is determined by metric *M*2. Network Parameters: *A* = Decay time constant at GABAergic I-to-E synapses, *B* = Decay time constant at GABAergic I-to-I synapses, *C* = Reduction of number of GABAergic I-to-E connections, *D* = Reduction of number of GABAergic I-to-I connections, *E* = Change of weight at GABAergic I-to-E connections, *F* = Change of weight at GABAergic I-to-I connections. Inset displays mean information (± standard deviation) for different numbers of parameters.

**Figure 4:**
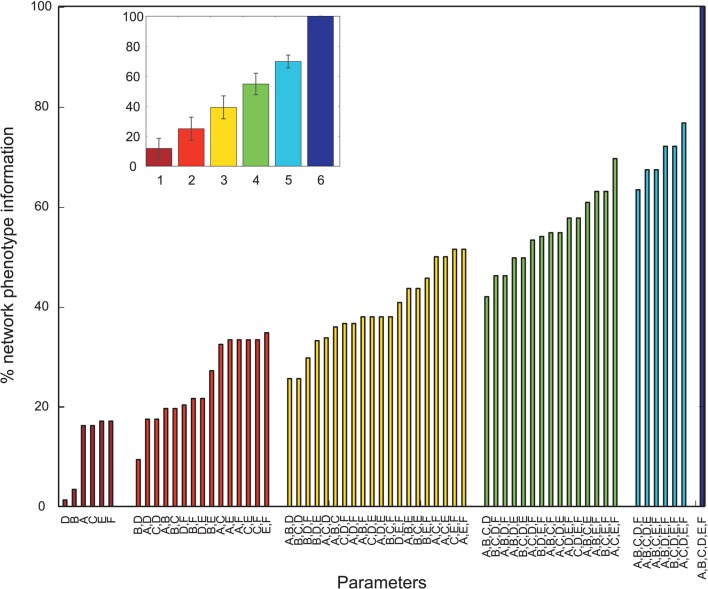
**Mutual information shared by single network parameters or combinations thereof with the phenotype of the network**. Phenotype is determined by metric *M*3. Network Parameters: *A* = Decay time constant at GABAergic I-to-E synapses, *B* = Decay time constant at GABAergic I-to-I synapses, *C* = Reduction of number of GABAergic I-to-E connections, *D* = Reduction of number of GABAergic I-to-I connections, *E* = Change of weight at GABAergic I-to-E connections, *F* = Change of weight at GABAergic I-to-I connections. Inset displays mean information (± standard deviation) for different numbers of parameters.

**Figure 5:**
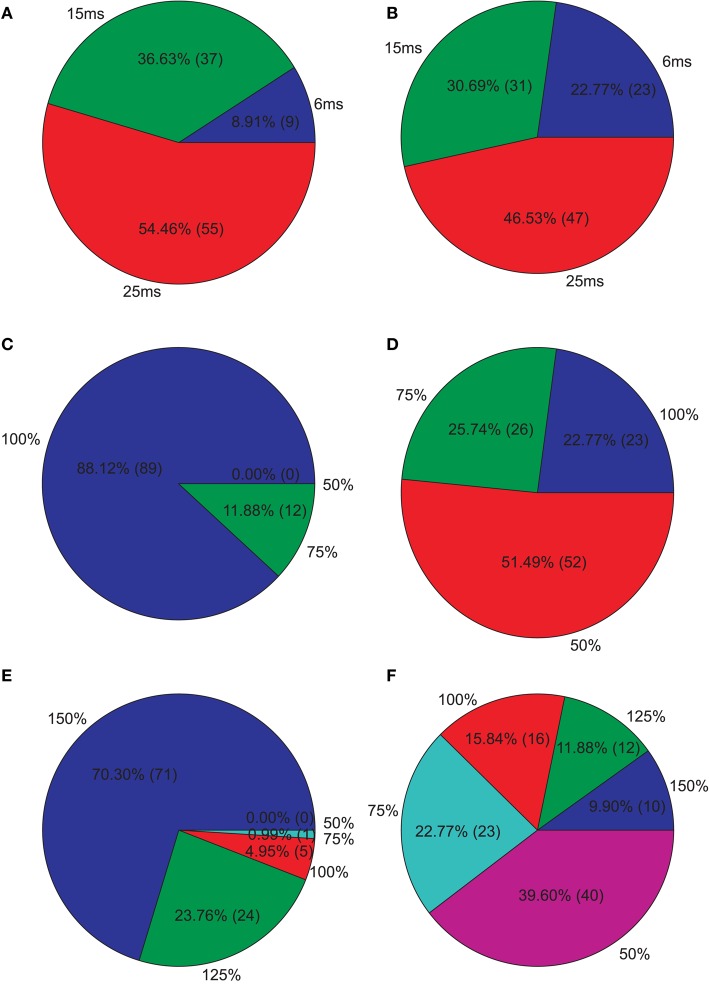
**Isolated network parameters : Pie charts showing the distribution of the 5% PCs having the highest M1 scores across the different instances of each parameter**. **(A)** GABAergic decay time constant at I-to-E synapses τ_*ie*_, **(B)** GABAergic decay time constant at I-to-I synapses τ_*ii*_, **(C)** Percentage of remaining I-to-E connections *r*_*ie*_, **(D)** Percentage of remaining I-to-I connections *r*_*ii*_, **(E)** Weight increase/decrease at I-to-E synapses *w*_*ie*_, **(F)** Weight increase/decrease at I-to-I synapses *w*_*ii*_.

Overall, many PVs produced oscillatory dynamics leading to high values for a given metric. However, no obvious single parameter describing circuit abnormalities could be identified by exploration of the data. In order to more formally quantify the information single parameters and combinations of single parameters had on the network phenotype, we calculated the normalized mutual information (nMI) as detailed in the Materials and Methods Section (see Figures 2–5). The average nMI for single parameters and for combinations of two or three parameters was quite low, although it was higher for *M*_2_ and *M*_3_ than for *M*_1_. This means that we could not identify one or two single parameters that led to SZ-like behavior in neither case. For metric *M*_2_ there is one single parameter with a high nMI (~40%) with phenotype, the GABAergic decay time constant at I-to-E synapses. This stems from the fact that for prolonged time constants the network failed to produce 30 Hz power (for 30 Hz drive) within the control range for most cases and thus did not show an SZ-like behavior.

#### 3.2.2. Hypothesis II

Our second hypothesis was that, the exact definition of SZ-like, would strongly influence the location of the SZ-region in parameter space. To this end, we defined three different illness metrics, which gradually incorporate more features of experimental results (see Materials and Methods).

Since we were interested in PVs that produce strong reductions, we further analyzed the PVs with the 5% highest M1. Figure 5 shows how these PVs distribute across the different instances of each parameter. We clearly see that longer decay times, especially at I-to-E synapses, leads to strong reductions. Furthermore, most PVs show an intact I-to-E connectivity and intact or even increased I-to-E weights. I-to-I connectivity and weight strength, however, can take all instances and typically seems to be intact to reduced in PVs showing strong reductions.

**Figure 6:**
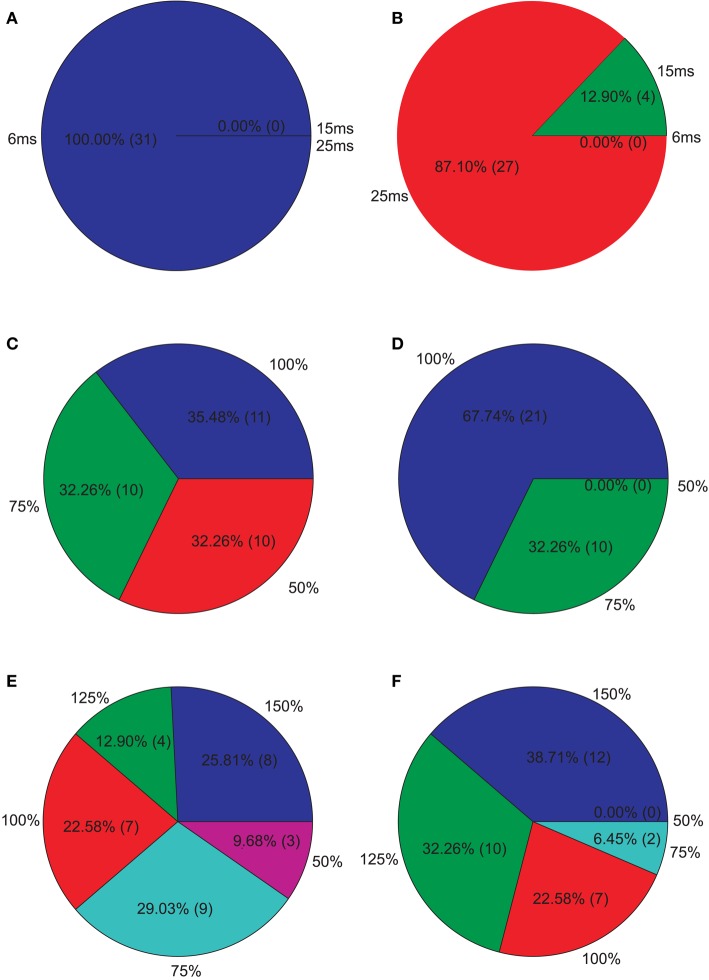
**Isolated network parameters : Pie charts showing the distribution of the 5% PCs having the highest M2 scores across the different instances of each parameter**. **(A)** GABAergic decay time constant at I-to-E synapses τ_*ie*_, **(B)** GABAergic decay time constant at I-to-I synapses τ_*ii*_, **(C)** Percentage of remaining I-to-E connections *r*_*ie*_, **(D)** Percentage of remaining I-to-I connections *r*_*ii*_, **(E)** Weight increase/decrease at I-to-E synapses *w*_*ie*_, **(F)** Weight increase/decrease at I-to-I synapses *w*_*ii*_.

However, we found that almost all of the above mentioned parameter combinations also produced power values in the 30 Hz band (in response to 30 Hz drive) that were substantially different from the control network, i.e., ha. Therefore, we analyzed which parameter combinations produced power values at 30 Hz in the 30 Hz drive condition that were within three standard deviations of the control power in that condition. We found that 31.06% (629/2025) parameter combinations satisfied this condition (the PVs satisfying this condition are hereafter referred to as *valid* vectors). We found that most valid parameter combinations produce a reduction in the 40 Hz component, i.e., a high M2 value. Now the parameter combinations showing the strongest reduction lay in a totally different region of the parameter space. Figure 6 again shows how these PVs distribute across the different instances of each parameter. We found that all PVs showing a “valid” response and a strong reduction in the 40 Hz component had an unchanged GABAergic decay time at inhibitory-to-excitatory synapses, but now had strongly prolonged GABAergic decay times at inhibitory-to-inhibitory synapses. Furthermore, an unchanged or only slightly reduced inhibitory-to-inhibitory connectivity seemed crucial for producing a “valid” response together with a strong reduction in the 40 Hz component. I-to-I connectivity tended to be reduced but with normal or stronger weights. I-to-E connectivity Furthermore, the strongest reduction produced by “valid” PVs was not as strong as those produced by the strongest not valid PVs (strongest valid PV:34.49% reduction, strongest overall PV: 48.29%).

**Figure 7:**
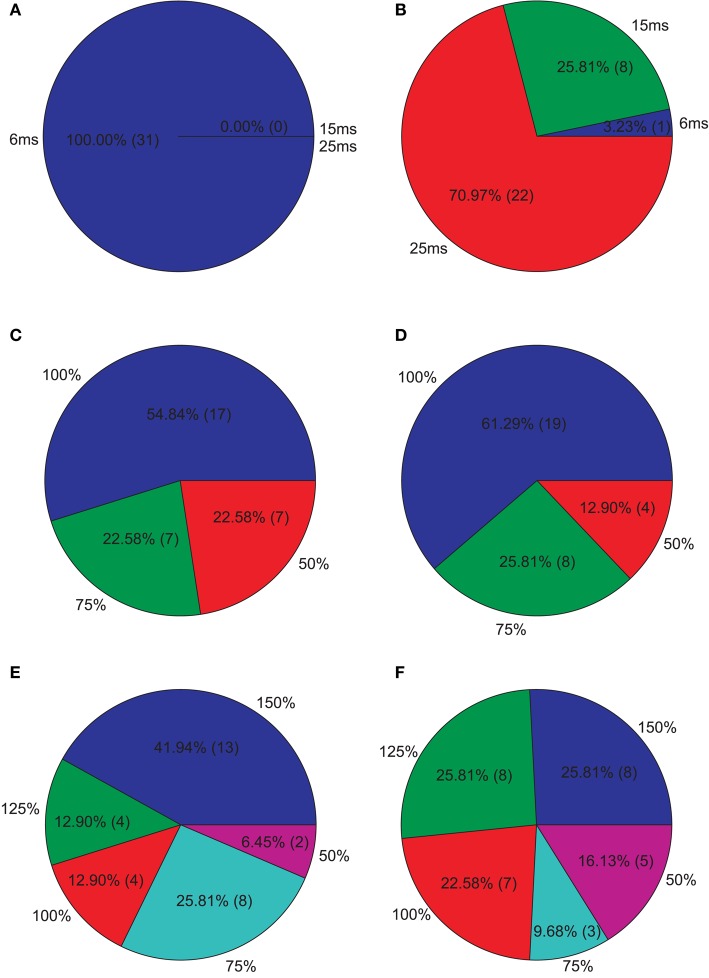
**Isolated network parameters : Pie charts showing the distribution of the 5% PCs having the highest M3 scores across the different instances of each parameter**. **(A)** GABAergic decay time constant at I-to-E synapses τ_*ie*_, **(B)** GABAergic decay time constant at I-to-I synapses τ_*ii*_, **(C)** Percentage of remaining I-to-E connections *r*_*ie*_, **(D)** Percentage of remaining I-to-I connections *r*_*ii*_, **(E)** Weight increase/decrease at I-to-E synapses *w*_*ie*_, **(F)** Weight increase/decrease at I-to-I synapses *w*_*ii*_.

Afterwards, we examined the top 5% of the PVs showing the highest M3 values, i.e., those valid PVs showing strong 40 Hz reduction and 20 Hz increase. Overall, we found unchanged I-to-E decay times and in an increase in I-to-I decay times for most PVs (Figure 7). Furthermore, most PVs tended to have unchanged connectivity (Figure 7).

#### 3.2.3. Hypothesis III

In order to test our third hypothesis, that different SZ-regions might show different network dynamics than the control network and than other SZ-regions, we selected 3 specific PVs for further analysis. The selected PVs were: (1) the PV showing the strongest reduction in 40 Hz power for 40 Hz drive (PV_*M*_1__), (2) the PV showing the strongest reduction in 40 Hz power for 40 Hz drive while giving a valid response to 30 Hz drive (PV_*M*_2__), and (3) the PV showing the strongest combined reduction at 40 Hz and increase at 20 Hz for 40 Hz drive (PV_*M*_3__). In detail, the selected parameter vectors were:

- PV_*M*_1__ = [τ_*ie*_ = 25 ms; τ_*ii*_ = 15 ms; *n*_*ie*_ = 100%; *n*_*ii*_ = 50%; *w*_*ie*_ = 150%; *w*_*ii*_ = 75%]
- PV_*M*_2__ = [τ_*ie*_ = 6 ms; τ_*ii*_ = 25 ms; *n*_*ie*_ = 100%; *n*_*ii*_ = 100%; *w*_*ie*_ = 75%; *w*_*ii*_ = 125%]
- PV_*M*_3__ = [τ_*ie*_ = 6 ms; τ_*ii*_ = 6 ms; *n*_*ie*_ = 100%; *n*_*ii*_ = 50%; *w*_*ie*_ = 150%; *w*_*ii*_ = 50%]

In order to test the first part of hypothesis III, we simulated network behavior 20 subjects for each of the selected PVs by generating different seeds for the random generator, thus altering specific connectivity but preserving connectivity statistics. In order to test the differences between the control group and the three different “schizophrenic” groups, we ran mixed model ANOVAs with GROUP (control, schizophrenic-M_*i*_) as a between subjects factor and POWER (40 and 20 Hz power at 40 Hz drive; and 30 Hz power at 30 Hz drive) as a repeated measures factor.

**Figure 8:**
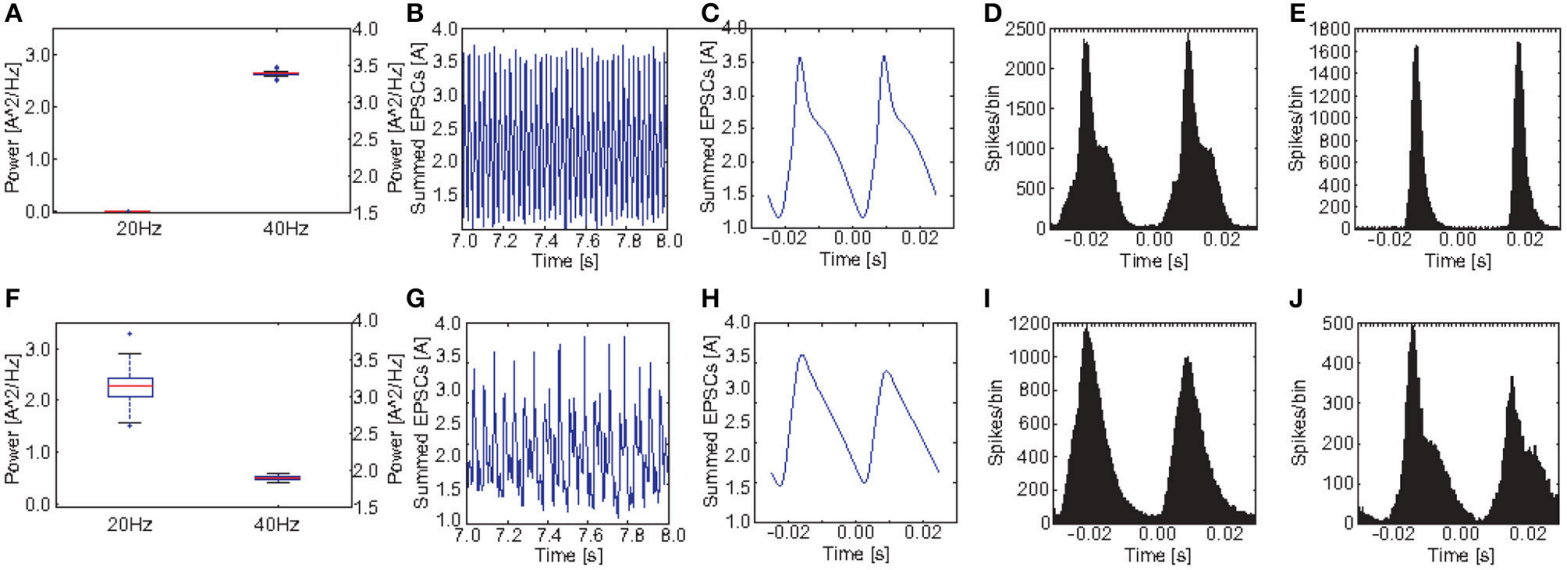
**Comparison of oscillatory activity between simulated control subjects and schizophrenic patients (as defined by metric M_**1**_)**. **(A,F)** The power in the 20 and 40Hz band for the 20 control subjects and the 20 schizophrenic patients, respectively. **(B,G)** A representative 0.5 s frame from the simulated raw EEG signal for simulated controls and schizophrenic patients, respectively. **(C,H)** Stimulus-locked EEG (i.e., EEG signal averaged over two consecutive cycles) for simulated controls and schizophrenic patients, respectively. **(D,I)** Stimulus-locked spike histogram of the pyramidal cell population for simulated controls and schizophrenic patients, respectively. **(E,J)** Stimulus-locked spike histogram of the basket cell population for simulated controls and schizophrenic patients, respectively. All plots depict results from 40 Hz stimulation trials.

Figures 8A,F show a comparison of the power in the 40 and the 20 Hz band for a stimulation with 40 Hz click trains between the control group and the *PV*_*M*_1__ group. Obviously both, the reduction at 40 Hz and the increase at 20 Hz, were a robust phenomenon. Interestingly, although there is a clear increase at 20 Hz for each of the 20 *PV*_*M*_1__ subjects, the variance was very high. The ANOVA showed that both the main effects of GROUP [*F*_(1, 38)_ = 667.56, *p* < 0.001] and POWER [*F*_(1.04, 39.40)_ = 4890.90, *p* < 0.001, Greenhouse-Geisser correction: ϵ = 0.518] were highly significant. Furthermore, there was a highly significant interaction GROUP^*^POWER [*F*_(1.04, 39.40)_ = 2987.92, *p* < 0.001, Greenhouse-Geisser correction: ϵ = 0.518]. This clearly demonstrates that control and schizophrenic groups produce very different oscillatory dynamics at 40 Hz drive.

**Figure 9:**
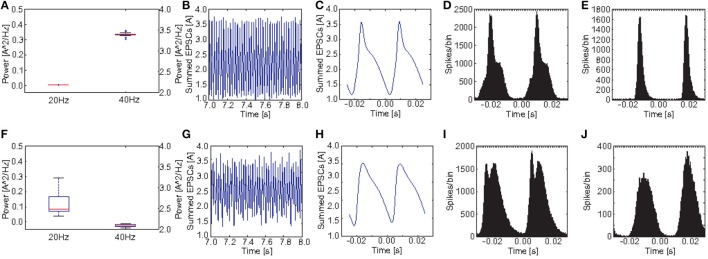
**Comparison of oscillatory activity between simulated control subjects and schizophrenic patients (as defined by metric M_**2**_)**. **(A,F)** The power in the 20 and 40Hz band for the 20 control subjects and the 20 schizophrenic patients, respectively. **(B,G)** A representative 0.5 s frame from the simulated raw EEG signal for simulated controls and schizophrenic patients, respectively. **(C,H)** Stimulus-locked EEG (i.e., EEG signal averaged over two consecutive cycles) for simulated controls and schizophrenic patients, respectively. **(D,I)** Stimulus-locked spike histogram of the pyramidal cell population for simulated controls and schizophrenic patients, respectively. **(E,J)** Stimulus-locked spike histogram of the basket cell population for simulated controls and schizophrenic patients, respectively. All plots depict results from 40 Hz stimulation trials.

Figures 9A,F show a comparison of the power in the 40 and the 20 Hz band for a stimulation with 40 Hz click trains between the control group and the *PV*_*M*_2__ group. Again we see a clear reduction at 40 Hz and an increase at 20 Hz, however, the increase was much less pronounced than before for M_1_. The ANOVA revealed that both the main effects of GROUP [*F*_(1, 38)_ = 8771.45, *p* < 0.001] and POWER [*F*_(1.60, 60.89)_ = 156950.51, *p* < 0.001, Greenhouse-Geisser correction: ϵ = 0.801] were highly significant. Furthermore, there was a highly significant interaction GROUP^*^POWER [*F*_(1.60, 60.89)_ = 2618.20, *p* < 0.001, Greenhouse-Geisser correction: ϵ = 0.801]. Again, although the effect was weaker, both groups differed significantly in the produced oscillatory dynamics at a 40 Hz drive.

**Figure 10:**
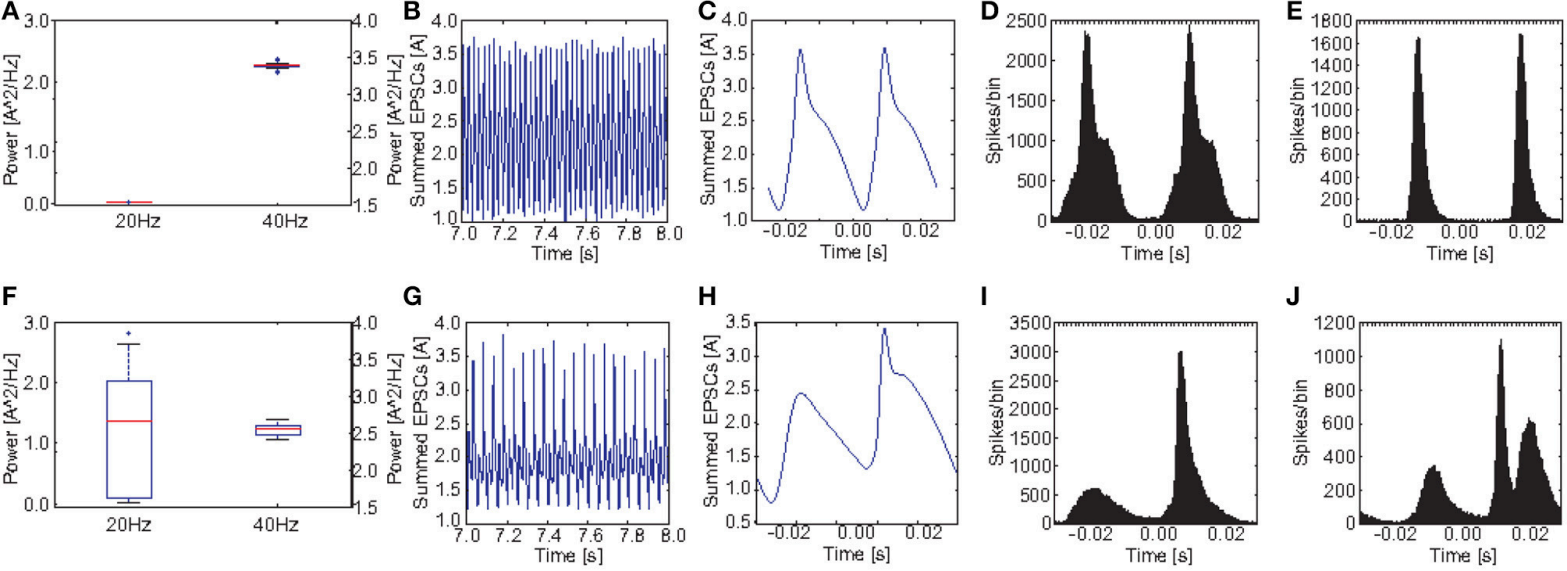
**Comparison of oscillatory activity between simulated control subjects and schizophrenic patients (as defined by metric M_**3**_)**. **(A,F)** The power in the 20 and 40Hz band for the 20 control subjects and the 20 schizophrenic patients, respectively. **(B,G)** A representative 0.5 s frame from the simulated raw EEG signal for simulated controls and schizophrenic patients, respectively. **(C,H)** Stimulus-locked EEG (i.e., EEG signal averaged over two consecutive cycles) for simulated controls and schizophrenic patients, respectively. **(D,I)** Stimulus-locked spike histogram of the pyramidal cell population for simulated controls and schizophrenic patients, respectively. **(E,J)** Stimulus-locked spike histogram of the basket cell population for simulated controls and schizophrenic patients, respectively. All plots depict results from 40 Hz stimulation trials.

Figures 10A,F show a comparison of the power in the 40 and the 20 Hz band for a stimulation with 40 Hz click trains between the control group and the *PV*_*M*_3__ group. Here we see that the difference at 40 Hz is even smaller than for M_2_ and that the power at 20 Hz is highly variable for the schizophrenic subjects. For some subjects there is a substantial increase compared to the healthy controls but for others there was no increase at all. Here the ANOVA showed that the main effect of GROUP [*F*_(1, 38)_ = 4.01, *p* = 0.052] was not significant, however the effect of POWER [*F*_(1.02, 38.77)_ = 1814.42, *p* < 0.001, Greenhouse-Geisser correction: ϵ = 0.510] was highly significant. Furthermore, there was a highly significant interaction GROUP^*^POWER [*F*_(1.02, 38.77)_ = 66.02, *p* < 0.001, Greenhouse-Geisser correction: ϵ = 0.510]. Here the ANOVA did not reveal a clear difference in oscillatory dynamics (although a trend toward significance was found; *p* = 0.052) between the control and the subject group.

In order to test the second part of hypothesis III and to better understand the mechanisms underlying these different oscillatory dynamics we investigated the simulated EEG signals and the spiking behavior of the cells in the model more closely. Again, we start with PV_*M*_1__.

Figure 8 shows a comparison of the power in both the 20 and 40Hz band, the raw signal as well as the EEG signal averaged over two continuous cycles of the 40 Hz stimulation (i.e., the signal divided in bins of two times the length of an oscillation/stimulation cycle aligned with the stimulation and the bins are then averaged. E.g., in case of a 40Hz drive, one cycle is 25ms long and each bin contains the signal from 25 ms before stimulation up to 25ms after the stimulation) together with the stimulus-locked spike time histograms of the excitatory and the inhibitory populations, in comparison to the control network. The raw EEG signal reveals that every second peak was suppressed in the schizophrenic network, explaining both, reduction and increase. We also see that stimulus-locked EEG and spike histograms became broader in the schizophrenic case, reflecting a worse entrainment at the driving frequency. Furthermore, we see that the first peak (for the stimulus-locked EEG and both spike histograms) was higher than the second, which explains the suppression of every second peak in the EEG.

We continue with PV_*M*_2__.

Figure 9, similar to Figure 8, shows a comparison of the power in both the 20 and 40Hz band, the raw signal as well as the EEG signal averaged over two continuous cycles of the 40 Hz stimulation together with the stimulus-locked spike time histograms of the excitatory and the inhibitory populations, in comparison to the control network, for PV_*M*_2__ this time. The raw EEG signal, as one would expect, did not show any beat skipping as before, but had lower amplitude and was more irregular than for the control network. Again, the cycle-averages show a strong broadening for both EEG signal and spike time histograms, which generally reflect a diminished ability to entrain to the gamma frequency stimulation.

Finally, we analyzed PV_*M*_3__. Note that the ANOVA before only showed a trend for differences in oscillatory dynamics.

Again as before, Figure 10 shows a comparison of the power in both the 20 and 40Hz band, the raw signal as well as the EEG signal averaged over two continuous cycles of the 40 Hz stimulation together with the stimulus-locked spike time histograms of the excitatory and the inhibitory populations, in comparison to the control network. As mentioned above the increase in 20 Hz power for the 20 schizophrenic subjects for this PV was highly variant. We chose to showcase a subject with a high increase in 20 Hz power in order to better visualize the changes in dynamics. Again, we see both the reduction at 40 Hz and the increase at 20 Hz clearly in the time-frequency analysis. In the raw EEG signal, both changes become instantly apparent, since the schizophrenic signal clearly showed the skipping of every other beat of the 40 Hz cycle. This is also reflected in stimulus-locked EEG as well as the stimulus-locked spike histograms. Excitatory cells almost only fired in the second cycle. Consequently, the inhibitory population also almost only fired in the second cycle. However, here we see a very interesting phenomenon: In the second cycle, there are two peaks of activity in the inhibitory population. The first one is very similar to the peak in the inhibitory population of the control network. The second peak however, was not seen in the control group at all. This peak leads to a suppression of activity in the excitatory population which lasted throughout the first cycle and thus caused the skipping of every other gamma cycle.

## 4. Discussion

### 4.1. Hypothesis I

Overall, we find that many different PVs can produce reductions in the gamma power in our 40 Hz entrainment paradigm and even many of them show valid 30 Hz entrainment and an increase in 20 Hz power. Furthermore, our information theoretic analysis revealed that single parameters only contain little information on the phenotype of the network. We found that several parameters had to be combined in order to obtain a substantial amount of phenotypic information. Thus, the EEG power abnormalities for entrainment found in schizophrenic patients are likely caused by an interaction of several mechanisms.

### 4.2. Hypothesis II

Our simulations demonstrate the most effective way to produce a marked decrease in 40 Hz power in response to 40 Hz drive, is to increase the inhibitory decay time constant τ_*ie*_ at inhibitory synapses at excitatory cells,while keeping I-to-E connectivity intact and strong (see Figure 5). This is most likely due to the fact that the oscillatory activity, which is produced by the excitatory postsynaptic currents (EPSCs), is most effectively controlled by inhibition onto excitatory cells. In more detail, the inhibitory neurons control excitatory firing by prohibiting pyramidal cell firing throughout most of the gamma cycle and only allowing firing during very brief time windows phase-locked to the gamma cycle. The decrease in gamma power seen for simulations where the τ_*ie*_ is prolonged is caused by pyramidal cells being inhibited for longer than one gamma cycle. Although, prolonging τ_*ie*_ is the most effective way to reduce gamma power it yields a strong decrease in 30 Hz power for 30 drive, which is not found experimentally (Kwon et al., 1999; Vierling-Claassen et al., 2008).

Our further analysis shows that an increase in τ_*ii*_ also leads to a strong decrease in gamma power, while affecting the response to 30 Hz drive much less than the aforementioned increase of τ_*ie*_. The effect of this prolonged inhibition between inhibitory neurons is, of course, strongest if the I-to-I connectivity is intact and strong (see Figures 6, 7). The prolonged inhibition of interneurons means that the interneurons are not able to entrain to the gamma rhythm which is fed forward to them by the sensory input and thus their control of the firing of the pyramidal cells is weaker, in turn leading to a weaker gamma rhythm of the excitatory network. Interestingly, while increasing the inhibitory time constant τ_*ie*_ also had a drastic effect on the response to 30 Hz drive, increasing τ_*ii*_ does not effect this response drastically. Interestingly, this motif of strong, prolonged I-to-I inhibition is present in both, the PVs having high M_2_ and the PVs having high M_3_ values, although they produce very different network dynamics. This suggests that strong, prolonged I-to-I inhibition selectively effects gamma entrainment, presumably by preventing interneurons to synchronize properly, while a change of τ_*ie*_ effects entrainment in a broader frequency range, presumably by affecting the imposition of the generated oscillation onto the pyramidal cells.

### 4.3. Hypothesis III

#### 4.3.1. SZ-like network dynamics differ from control network dynamics

Although the three SZ-like networks investigated in our study, produce very different raw EEG signals, they all strongly differ from the control network (see Figures 5A, 6A, 7A) Furthermore, they alter the EEG signal by either suppressing responses during specific cycles of oscillations or by decreasing the response amplitude.

#### 4.3.2. SZ-like network dynamics from each other

We also found, that the oscillatory dynamics of the three tested networks are very different from each other. While PV_*M*_1__ shows a mixture of reduced EEG signal amplitude and suppression of specific oscillation cycles (see Figure 8), PV_*M*_2__ only shows a reduction in signal amplitude (see Figure 9), and PV_*M*_3__ only shows suppression of responses in every other oscillation cycle (see Figure 10).

Since PV_*M*_1__, characterized by a prolongation of strong I-to-E inhibition. also led to a very strong reduction of power in 30 Hz stimulation trials, which is not seen in experiments, it is unlikely that this mechanism plays an important role in gamma deficits in schizophrenic patients. However, it highlights that models of gamma entrainment should not solely focus on the reduction of gamma power as an indicator for SZ-like behavior. Future models will have to explain more features of the experimental gamma entrainment data, such as unchanged 30 Hz power and increased 20 Hz power.

The fundamental difference between the dynamics produced by PV_*M*_2__ and PV_*M*_3__ is very interesting, since the only difference in these network responses is the existence or absence of an increase in power at 20 Hz. An increase in power at 20 Hz is found experimentally (Kwon et al., 1999; Vierling-Claassen et al., 2008), however it is not as prominent as the gamma reduction. Notably, the 20 subjects simulated using PV_*M*_3__ showed a very high variance in 20 Hz power, ranging from strong increases in power to no change at all (see Figure 10F). Further investigations should be made to identify the reasons underlying the high variance found in this SZ-like group.

### 4.4. Limitations

As has been argued elsewhere (e.g., Siekmeier and vanMaanen, 2013), although the deficit in gamma entrainment is not a core symptom of schizophrenia, it presents an ideal target for computational modeling studies like ours. First, the above mentioned shift toward endophenotypic measures of psychiatric disorders, produces an increasing amount of experimental data, which show that abnormalities in the gamma band seem to be most consistent in schizophrenic patients. Second, gamma oscillations have been identified to underly several important computations in many different sensory and cognitive functions, which suggests that a deficit therein might explain many of the symptoms found in patients with schizophrenia.

Obviously, the network model used in our study has clear limitations and our approach only constitutes a first step toward the use of biophysically detailed models in computational psychiatry. Therefore, we want to discuss these limitations here to show how the proposed approach can be extended in future work.

The network model does not contain several components that are likely to influence oscillatory dynamics *in vivo*. First, there are no NMDA receptors in the model, however, these are likely to be important in the etiology of schizophrenia (e.g., Lisman et al., 2008) and have been demonstrated to influence gamma oscillations (e.g., Kirli et al., 2014; Jadi et al., 2016). Interestingly, these studies both have shown that in this case single perturbations to the NMDA receptors can account for gamma oscillation deficits. Therefore, it will be important to include NMDA receptors to the proposed model and to explore the interactions of NMDA receptor deficits with the abnormalities modeled here. Kömek et al. (2012) found changes in network oscillations in response to auditory entrainment stimulation by modulating the excitability of fast-spiking interneurons via changes in dopaminergic drive. Taken together, these findings suggest that in some cases single perturbation can potentially account for experimental findings whereas in other cases several perturbations have to coincide. Given the enormous heterogeneity of schizophrenia and the existence of different subpopulation of patients, it will be very interesting to explore the differences in auditory entrainment in these subpopulations in much more detail. It might be the case that a mapping of these subpopulations to the different cases outlined above exists.

Second, no distinction of different interneuron types was included in the model. However, Chandelier cells (a PV^+^ subtype that only targets pyramidal cell axon initial segments), for example, are hypothesized to play a crucial role in schizophrenia (Vierling-Claassen et al., 2008; Vierling-Claassen and Kopell, 2009), although there is some controversy (see e.g., Gonzalez-Burgos and Lewis, 2008, 2012).

Furthermore, no interneuron-targeting inhibitory, calretinin positive (CR^+^) neurons were included, although dis-inhibition by altering CR^+^ neurons has been shown to contribute to SZ-like behavior in working memory models (Wang et al., 2004). We also did not include LTS, dendrite-targeting inhibitory neurons, although they have a pronounced influence on low frequency entrainment (Vierling-Claassen et al., 2010) and thus might play a role for the changes in the beta range. Gap junctions between interneurons were omitted as well although they have been shown to be powerful synchronizers in hippocampal networks (Bartos et al., 2007). Hyperpolarizing vs. shunting inhibition (or even depolarizing inhibition) has not been addressed although it can potentially modulate oscillation frequencies (Vida et al., 2006; Woodruff et al., 2009, 2010, 2011).

In addition to these synaptic/network level components, there is also evidence that genetic alterations in schizophrenic patients affect cell-intrinsic properties that influence cell excitability and thus could potentially change the oscillatory dynamics of the network. In a recent modeling study, (Mäki-Marttunen et al., 2016) show that the interaction of small changes to parameters regulating ion channels and internal calcium concentrations in cortical pyramidal cells, can drastically change the excitability of the cells. The novelty of their approach is, that the parameter changes implemented in their model are directly derived from susceptibility genes identified from a large genome-wide association study (Ripke et al., 2014). An inclusion of these cell-intrinsic into our current model is ongoing work in our group.

Although, we tried to incorporate more detail of the experimental results from the EEG/MEG studies used in this work, we have not taken into account the differences between healthy controls and schizophrenic patients when stimulated in the beta range (i.e., 20 Hz stimulation). Furthermore, we have not looked at other frequency ranges outside of the gamma and beta range, although differences have been reported in other ranges (e.g., alpha range or high-gamma Uhlhaas and Singer, 2010). An exploration of these other frequency ranges would have further increased the number of simulations. Furthermore, we strongly expect that extending the model to incorporate more of the above-mentioned components would be necessary to change the model presented to explain these other phenomena mentioned here and was therefore beyond the scope of this article.

Spencer (2009) have explored similar network level perturbations in the context of induced, rather than evoked, gamma oscillations, and find strong influences on oscillation power and overall synchrony. Therefore, it would be interesting to complement our present analysis with an exploration of induced gamma oscillations.

Generally, we think that the approach outlined here, should be extended to produce a more detailed, in both, the biological detail represented by the model and also the detail of experimental data reproduced by the model. This would greatly increase the explanatory power of the insights gained through this modeling effort. Additionally, the process itself will likely shed light onto the mechanisms underlying oscillatory deficits in schizophrenic patients and produce novel hypotheses that can be experimentally tested.

## 5. Conclusion

The development of new medication for the treatment of psychiatric disorders such as schizophrenia has met with limited success over the past decades. Many have argued that a shift toward endophenotypic measures would provide clearer mappings between these measures and the underlying genetic alterations, ultimately facilitating this drug development. As Siekmeier points out (Siekmeier, 2015), detailed computational models of endophenotypic measures can provide a crucial tool in such an effort. However, often computational modeling efforts seem to neglect the multifactorial nature of these system level measures and only investigate one specific mechanisms that might produce abnormal results, without exploring the many other ways which could possibly produce the same abnormality (Pavão et al., 2015).

Here we provide an underpinning of the importance of a multifactorial view when modeling endophenotypic measures. Furthermore, we demonstrate how an exhaustive exploration of the parameter space of such a model can be used to extract information on the different mechanisms that might underly abnormalities in schizophrenic patients. We also find that possible mechanisms depend on the amount of experimental detail which is incorporated in the analysis.

In conclusion, we have presented an biophysically detailed implementation of a biomarker of schizophrenia, which can serve as a basis for the exploration of mechanisms underlying oscillatory deficits in schizophrenia and as a tool for the identification and testing of novel mechanisms of action for anti-psychotic drugs.

## Author contributions

Authors CM, AS, and BZ jointly conceived the study. CM implemented all changes to the original network model used here, implemented and performed all parameter searches and all analyses in this work. CM wrote the manuscript with supervision from AS and BZ.

### Conflict of interest statement

The authors declare that the research was conducted in the absence of any commercial or financial relationships that could be construed as a potential conflict of interest.
